# Interferon‐stimulated gene 56 positively regulates Toll‐like receptor 3‐mediated CXCL10 expression in human renal proximal tubular epithelial cells

**DOI:** 10.1002/2211-5463.13851

**Published:** 2024-06-23

**Authors:** Mayuki Tachizaki, Sho Sakamoto, Yuri Kobori, Yoshiya Asano, Shogo Kawaguchi, Kazuhiko Seya, Hiroshi Tanaka, Eiji Morita, Tadaatsu Imaizumi

**Affiliations:** ^1^ Department of Vascular and Inflammatory Medicine Hirosaki University Graduate School of Medicine Japan; ^2^ Department of Biochemistry and Molecular Biology Hirosaki University Faculty of Agriculture and Life Science Japan; ^3^ Department of Respiratory Medicine Hirosaki University Graduate School of Medicine Japan; ^4^ Department of Neuroanatomy, Cell Biology and Histology Hirosaki University Graduate School of Medicine Japan; ^5^ Department of School Health Science Hirosaki University Faculty of Education Japan

**Keywords:** C‐X‐C motif chemokine ligand 10, human renal proximal tubular epithelial cell, interferon‐stimulated gene 56, interferon‐β, Toll‐like receptor 3

## Abstract

Viral infections in tubular epithelial cells lead to the production of inflammatory cytokines by innate immunity, causing tubulointerstitial nephritis. TLR3 recognizes viral infections and acts via the activation of interferon (IFN)/IFN‐stimulated genes (ISGs). This study investigates the role of ISG56, a representative ISG, in TLR3 signaling in cultured human renal proximal tubular epithelial cells (hRPTECs). To this end, hRPTECs were stimulated by a synthetic TLR3 ligand, polyinosinic‐polycytidylic acid (poly IC), recombinant human interferon‐β [r(h)IFN‐β] or Japanese encephalitis virus (JEV) infection and assayed for inflammatory cytokine mRNA expression by RT‐qPCR, and protein expression via western blotting or ELISA. ISG56 was expressed by poly IC or r(h)IFN‐β and IFN‐β knockdown reduced poly IC‐induced expression of ISG56 and CXCL10. Moreover, ISG56 knockdown reduced poly IC‐ or r(h)IFN‐β‐induced expression of CXCL10 at the same time as increasing JEV growth and reducing CXCL10 expression induced by JEV infection. Overall, TLR3 signaling induced IFN‐β‐dependent expression of ISG56 and CXCL10. We show that ISG56 possibly plays a critical role in antiviral immunity of hRPTECs by positive regulation of IFN‐β‐mediated CXCL10 expression downstream of TLR3.

## Graphical abstract

Viral infection in tubular epithelial cells causes tubulointerstitial nephritis, also activating innate immunity. This study investigates the role of interferon‐stimulated gene 56 (ISG56), one of the ISGs that suppress viral replication, downstream of Toll‐like receptor 3 (TLR3), a receptor that recognizes viral infection, in cultured human proximal tubular epithelial cells (hRPTECs). ISG56 positively regulated IFN‐β‐mediated C‐X‐C motif chemokine ligand 10 (CXCL10) expression downstream of TLR3 in human renal proximal tubular epithelial cells.

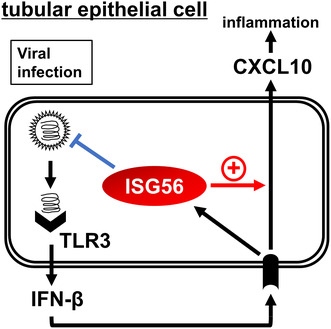

**Abbreviations**
AKI: acute kidney injury
CXCL: C‐X‐C motif chemokine ligand
CXCR: C‐X‐C motif chemokine receptor
dsRNA: double‐stranded RNA
hRPTEC: human renal proximal tubular epithelial cell
IFIT1: interferon‐induced protein with tetratricopeptide repeat 1
IFN: interferon
ISG: interferon‐stimulated gene
JEV: Japanese encephalitis virus
MOI: multiplicity of infection
PB: phosphate buffer
PBS: phosphate‐buffered saline
poly IC: polyinosinate polycytidylate
PRR: pattern recognition receptor
r(h)IFN‐β: recombinant human interferon‐β
RT‐qPCR: reverse transcriptase‐quantitative PCR
SEM: scanning electron microscopy
siRNA: small interfering RNA
STAT1: signal transducer and activator of transcription 1
TLR: Toll‐like receptor

Viral kidney infections include glomerulonephritis and interstitial nephritis [1]. Interstitial nephritis occurs in renal transplant recipients and in patients with SARS‐CoV‐2 infection. Renal transplant patients are prone to immunocompromised states triggered by immunosuppressive drugs, inflammation or tissue injury, leading to viral reactivation [2]. Cytomegalovirus and BK virus are latent in tubular epithelial cells of the host or graft, and their reactivation can cause tubulointerstitial nephritis [3]. Also, a recent pandemic of SARS‐CoV‐2 infection reportedly causes tubulointerstitial damages because SARS‐CoV‐2 infects tubular epithelial cells directly [4]. Although several viruses are known to infect tubular epithelial cells, the antiviral immune system in tubular epithelial cells remain largely unknown, and no specific treatment is available for viral kidney infections. To establish a new treatment strategy, the details of the defense mechanisms against viral kidney infections need to be elucidated.

Host cells possess pattern recognition receptors (PRRs) that recognize viral and microbial infections. PRRs sense pathogen‐associated molecular patterns and activate immunity, and consequently the activation of inflammatory pathways [5]. PRRs include Toll‐like receptors (TLRs); among these, TLR3 is characterized by recognition of virus‐derived double‐stranded RNA (dsRNA) on the surface of endosomes [5]. TLR3 activates interferon regulatory factor 3 and nuclear factor‐κB, which leads to the production of type I interferon (IFN) (IFN‐α and IFN‐β) and inflammatory cytokines [6]. IFN‐α is mainly produced by immune cells, whereas IFN‐β is mainly expressed by epithelial cells, endothelial cells, fibroblasts, etc. IFNs act on cells via autocrine or paracrine signaling to induce IFN‐stimulated genes (ISGs). ISGs are involved in many processes against viral infections and activate immune and inflammatory responses [7].

To protect against viral infection, it is important to prevent viral replication and transmission of the virus to other cells. ISG56, a member of the ISGs family, encodes a protein with tetratricopeptide repeat, and is also called IFN‐induced protein with tetratricopeptide repeat 1 (IFIT1) [8]. The ISG56/IFIT1 protein is an important protein in antiviral innate immune response because it prevents the spread of viral infection by inhibiting translation and replication [8]. In a previous study using human mesangial cells [9] and glomerular endothelial cells [10], ISG56 was induced by TLR3 signaling and is related to C‐X‐C motif chemokine ligand 10 (CXCL10) expression. Tubular epithelial cells transport water and solutes during urine production. These cells also play crucial roles in the immunological reactions [11]. It has been shown that tubular epithelial cells express TLR3, and activation of TLR3 induces the production of tumor necrosis factor‐α and IFN‐β [12, 13]. These reports suggest that these cells possess the ability to induce antiviral response. However, there are no reports regarding the expression and the functional roles of ISG56 in tubular epithelial cells.

CXCL10, a chemokine originally named IFN‐γ‐inducible protein‐10, is secreted not only by immune cells, but also by epithelial cells, endothelial cells and fibroblasts under IFN stimulation [14]. The binding of CXCL10 to the C‐X‐C motif chemokine receptor 3 (CXCR3) on immune cells promotes lymphocyte activation and migration at the site of infection [14]. Additionally, the CXCL10‐CXCR3 system is associated with direct antiviral activity against some RNA viruses [15]. It has been reported that stimulation with IFN‐γ, tumor necrosis factor‐α and heme oxygenase‐1 increases CXCL10 expression in tubular epithelial cells [13, 16]. However, there are no reports that TLR3 signaling triggers CXCL10 expression in tubular epithelial cells.

Polyinosinic‐polycytidylic acid (poly IC) is a synthetic analog that imitates the structure of viral dsRNA and activates TLR3 [17]. Viral infection can be mimicked by stimulating cells with poly IC. This study examined ISG56 expression and its role in CXCL10 expression upon stimulation with poly IC in cultured human renal proximal tubular epithelial cells (hRPTECs). In addition, the expression and antiviral role of ISG56 in hRPTECs infected with Japanese encephalitis virus (JEV), the positive stranded RNA virus, were also investigated.

## Materials and methods

### Cell culture and virus

The hRPTECs were obtained from Lonza (Walkersville, MD, USA). The REGM BulletKit (Lonza) was used to culture the cells. Cultured cells were stimulated with poly IC (P9582; Sigma, St Louis, MO, USA) or recombinant human IFN‐β [r(h)IFN‐β] (PeproTech, Rehovot, Israel). In addition, the cells were infected with JEV strain AT31 (kindly provided by Eiji Konishi, Osaka University). Vero cells were cultured in Dulbecco's modified Eagle's medium containing 10% fetal bovine serum and 1% penicillin/streptomycin.

### Scanning electron microscopy (SEM)

hRPTECs were prefixed with 2% paraformaldehyde and 2.5% glutaraldehyde in 0.1 m phosphate buffer (PB). The cells were treated with 1% tannic acid in 0.1 m PB and postfixed with 1% osmic acid in 0.1 m PB. After dehydration with ethanol and treatment with t‐butyl alcohol, the cells were frozen at −20 °C. After lyophilization, the cells were coated with platinum of 100 Å thickness and observed with a scanning electron microscope (JSM‐6510LV; JEOL, Tokyo, Japan).

### Albumin uptake

hRPTECs were seeded and cultured in 35‐mm glass base dishes. The cells were incubated with BSA‐Alexa488 (A13100; Thermo Fisher Scientific, Waltham, MA, USA) for 6 h. After washing three times with phosphate‐buffered saline (PBS), Hoechst 33342 (Dojindo, Kumamoto, Japan) was used for nuclear staining. Laser scanning confocal microscopy (C1si laser microscope; Nikon, Tokyo, Japan) was used for observation.

### Small interfering RNA (siRNA)

Fifty nanomolar siRNAs against IFN‐β (as described in Imaizumi *et al*. [18] and S7188; Thermo Fisher Scientific), ISG56 (S100445879 and SI02660777; Qiagen, Hilden, Germany) or a non‐targeting negative control (1027281; Qiagen) were transfected into the cells cultured in antibiotic‐free medium using Lipofectamine RNAiMAX (Thermo Fisher Scientific) in accordance with the manufacturer's instructions. After medium changes and 24 h of incubation, poly IC (10 μg·mL^−1^), r(h)IFN‐β (0.1 ng·mL^−1^) or JEV [multiplicity of infection (MOI) = 1.0] was added to the medium.

### Pretreatment with fludarabine

The cells were pretreated with fludarabine (F9813; Sigma), an inhibitor of signal transducer and activator of transcription 1 (STAT1) activation, at 10 μm for 1 h, and then treated with 10 μg·mL^−1^ poly IC.

### Reverse transcriptase‐PCR (RT‐PCR) and RT‐quantitative PCR (RT‐qPCR)

Total RNA was extracted from cells using a NucleoSpin RNA kit (Macherey‐Nagel, Düren, Germany). The dNTP mix (Thermo Fisher Scientific) and M‐MLV reverse transcriptase (Thermo Fisher Scientific) were used to synthesize single‐stranded cDNA from total RNA. In RT‐PCR analysis, the cDNAs of cubilin, CD13 and GAPDH were amplified using specific primers and GoTag Green (Promega, Madison, WI, USA) for 40 cycles at 94 °C, 55 °C and 72 °C. PCR products were analyzed by electrophoresis on 1.8% agarose gels containing ethidium bromide. In RT‐qPCR analysis, specific primers and SsoAdvanced Universal SYBR Green Supermix (Bio‐Rad, Hercules, CA, USA) were used to quantify IFN‐β, ISG56, CXCL10 and GAPDH mRNA expression. PCR was performed by CFX real‐time PCR detection System (Bio‐Rad) for 40 cycles at 95 °C, 60 °C and 72 °C. GAPDH mRNA was used as an internal control. The average of the triplicate measurements in unstimulated cells was set to 1, and the values of ISG56 and CXCL1 are shown as fold increase compared to unstimulated cells. The values of CXCL10 and IFN‐β mRNA expression are presented as arbitrary units because these mRNAs were undetectable in untreated cells. The primers for IFN‐β, cubilin and CD13 were from Eurofins Genomics (Louisville, KY, USA), and others were from Fasmac (Atsugi, Japan).

The primer sequences are:

Cubilin‐F: 5′‐GCTCATCCAGGCTCCCGACTCTAC‐3′,
Cubilin‐R: 5′‐TTGAAGCCTGCCCTGGTTACACTG‐3′,
CD13‐F: 5′‐GTCTACTGCAACGCTATCGC‐3′,
CD13‐R: 5′‐GATGGACACATGTGGGCACCTTG‐3′,
GAPDH‐F (RT‐PCR): 5′‐CCACCCATGGCAAATTCCATGGCA‐3′,
GAPDH‐R (RT‐PCR): 5′‐TCTAGACGGCAGGTCAGGTCCACC‐3′,
ISG56‐F: 5′‐TAGCCAACATGTCCTCACAGAC‐3′,
ISG56‐R: 5′‐TCTTCTACCACTGGTTTCATGC‐3′,
IFN‐β‐F: 5′‐ACTGCCTCAAGGACAGGATG‐3′,
IFN‐β‐R: 5′‐AGCCAGGAGGTTCTCAACAA‐3′,
CXCL1‐F: 5′‐ATGGCCCGCGCTGCTCTCTCC‐3′,
CXCL1‐R: 5′‐GTTGGATTTGTCACTGTTCAG‐3′,
CXCL10‐F: 5′‐TTCAAGGAGTACCTCTCTCTAG‐3′,
CXCL10‐R: 5′‐CTGGATTCAGACATCTCTTCTC‐3′,
GAPDH‐F (RT‐qPCR): 5′‐GCACCGTCAAGGCTGAGAAC‐3′,
GAPDH‐R (RT‐qPCR): 5′‐ATGGTGGTGAAGACGCCAGT‐3′.

### Western blotting

After rinsing twice with PBS, cultured cells were dissolved in the Laemmli sample buffer. Twenty micrograms of protein was loaded on 5–20% polyacrylamide gel electrophoresis using e‐PAGEL (ATTO, Tokyo, Japan). The separated proteins were transferred from the gel to polyvinylidene difluoride membranes (Merck Millipore, Darmstadt, Germany), and the membranes were blocked with non‐fat milk at room temperature for 2 h. The membranes were then packed in an antibody solution and were incubated at 4 °C overnight. The following antibodies were used: rabbit anti‐TLR3 (dilution 1 : 1000; 6961S; Cell Signaling Technology, Danvers, MA, USA), rabbit anti‐ISG56 (dilution 1 : 1000; GTX118713; GeneTex, Irvine, CA, USA), rabbit anti‐STAT1 (dilution 1 : 7500; sc‐346; Santa Cruz Biotechnologies, Dallas, TX, USA), mouse anti‐phosphorylated STAT1 (dilution 1 : 3000; sc‐136229; Santa Cruz Biotechnologies) and rabbit anti‐actin IgG (dilution 1 : 3000; A5060; Sigma). After washing, the membranes were packed in horseradish peroxidase‐conjugated anti‐mouse IgG (Thermo Fisher Scientific) or anti‐rabbit IgG (Medical and Biological Laboratories, Nagoya, Japan) solution and were incubated at room temperature for 1 h. A Luminata Crescendo substrate (Merck Millipore) was used for immunodetection. Intensity of bands was quantified using the imagej (NIH, Bethesda, MD, USA).

### ELISA

ELISA kits for detecting CXCL10 and IFN‐β were obtained from R&D Systems (Minneapolis, MN, USA) and PBL Assay Science (Piscataway, NJ, USA), respectively, and the manufacturers' recommended protocols were followed.

### Immunofluorescence

The cells were seeded and incubated in gelatin‐coated glass base dishes. The cells were stimulated with poly IC for 24 h, fixed with 10% formaldehyde and permeabilized with 100% methanol at −20 °C. After blocking with 1% BSA‐PBS for 30 min, cells were incubated with rabbit anti‐ISG56 (dilution 1 : 100; GTX118713) overnight at 4 °C. After washing, cells were incubated with fluorescence‐labeled secondary anti‐rabbit antibody (ab150077; Abcam, Cambridge, UK) at room temperature for 1 h. Nuclei were stained with Hoechst 33342 (Dojindo) and visualized by laser scanning confocal microscopy (C1si laser microscope).

### Focus forming assay

The hRPTECs were infected with JEV (MOI = 1.0) and cultured for 24 or 48 h. The supernatant was collected and the viruses in the culture supernatants were inoculated to Vero cells. Two hours later, the methylcellulose was applied over the Vero cells, which were then incubated for an additional 48 h. Subsequently, the cells were fixed using 4% paraformaldehyde for 10 min, followed by three washes with PBS. The cells were incubated with rabbit anti‐JEV NS3C antibody (dilution 1 : 3000), which targets the recombinant C‐terminal region of the JEV NS3 protein (amino acids 1652–2093), in a solution containing 0.1% Triton X‐100 and 10% fetal bovine serum in PBS. This incubation was performed for 1 h at room temperature. Then, the cells were washed three times with PBS and incubated with an anti‐rabbit IgG R488 antibody (dilution 1 : 5000; Jackson ImmunoResearch, West Grove, PA, USA) for another 1 h at room temperature. The reaction was terminated by rinsing with Milli‐Q water (Merck Millipore). The visualized cells were counted, allowing for the calculation of viral infection titers.

### Statistical analysis

The results of the RT‐qPCR, ELISA and JEV titer are shown as the mean ± SD. Statistical analysis was performed using the Mann–Whitney *U*‐test. *P* < 0.05 was considered statistically significant.

## Results

### hRPTECs show characteristics of renal proximal tubular epithelial cells

**Fig. 1:**
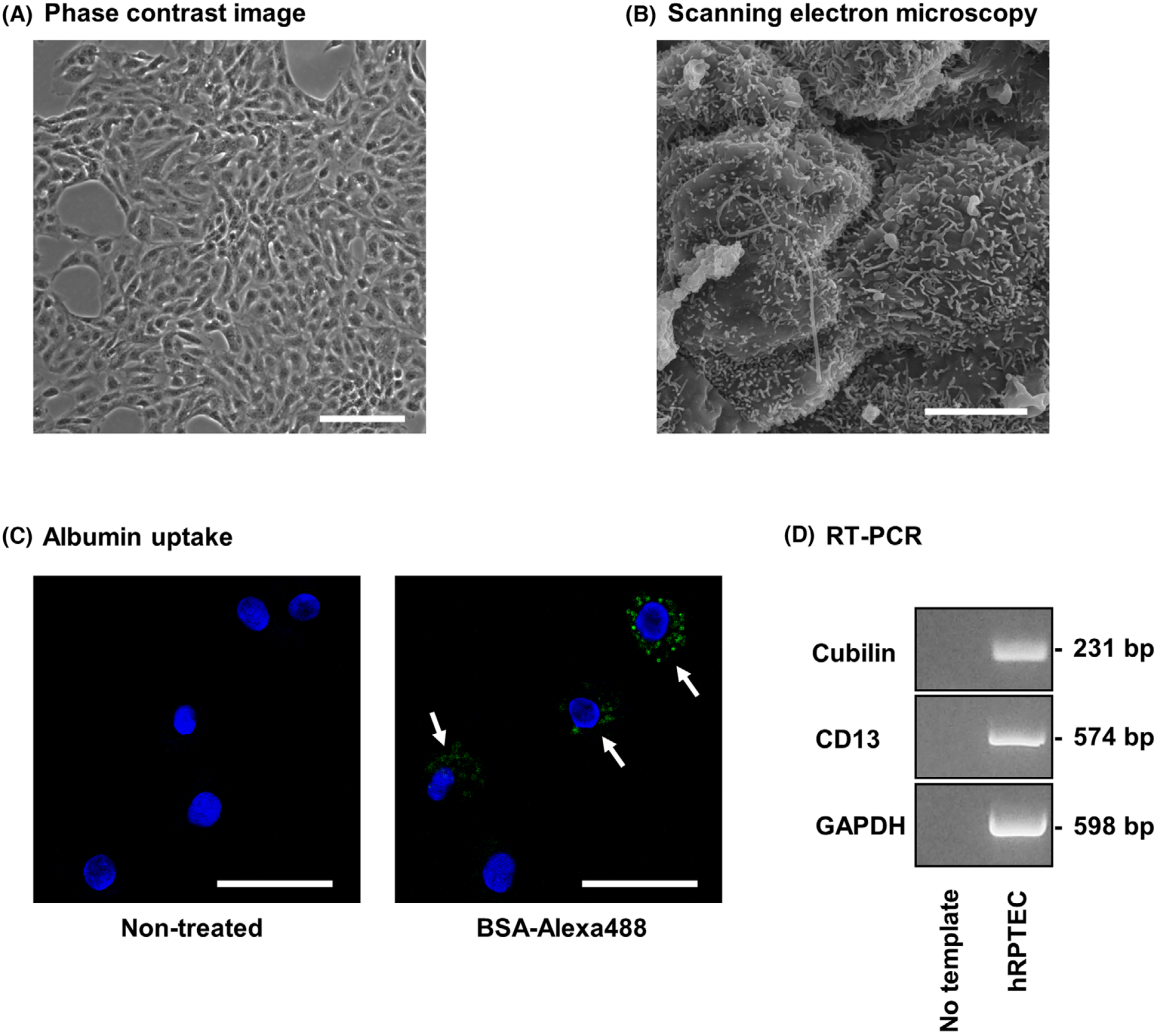
hRPTECs had characteristics of renal proximal tubular epithelial cells. (A) The cells were cultured and observed by phase contrast microscopy. The white scale bar represents 200 μm (*n* = 1). (B) The cells were seeded on a transwell. After 5 days, the cells were observed by scanning electron microscopy. The white scale bar represents 5 μm. (*n* = 1). (C) Cultured hRPTECs were treated with BSA‐Alexa488 (50 μg·mL^−1^) for 6 h, and Hoechst 3334 was used for nuclear staining. The white arrows represent the uptake of albumin‐Alexa488 by hRPTECs. The white scale bar represents 50 μm (*n* = 1). (D) RNA was extracted from the cells, and cDNA was synthesized from total RNA. Cubilin, CD13 and GAPDH cDNA were amplified by RT‐PCR, and the PCR products were subjected to agarose gel electrophoresis (*n* = 1).

The phase contrast imaging of hRPTECs used in this study revealed the typical monolayer (Fig. 1A). SEM showed that the cells had dense microvilli forming a brush border (Fig. 1B). When BSA‐Alexa488 was added to the culture medium, uptake of albumin‐Alexa488 by the cells was observed (Fig. 1C). RT‐PCR revealed that hRPTECs expressed cubilin, which acts as an endocytic receptor for the intrinsic factor‐Vitamin B12 complex, and CD13 (also named as aminopeptidase N), which is known to be expressed in microvilli of renal proximal tubular epithelial cells (Fig. 1D).

### ISG56 and IFN‐β are expressed by poly IC in hRPTECs

**Fig. 2:**
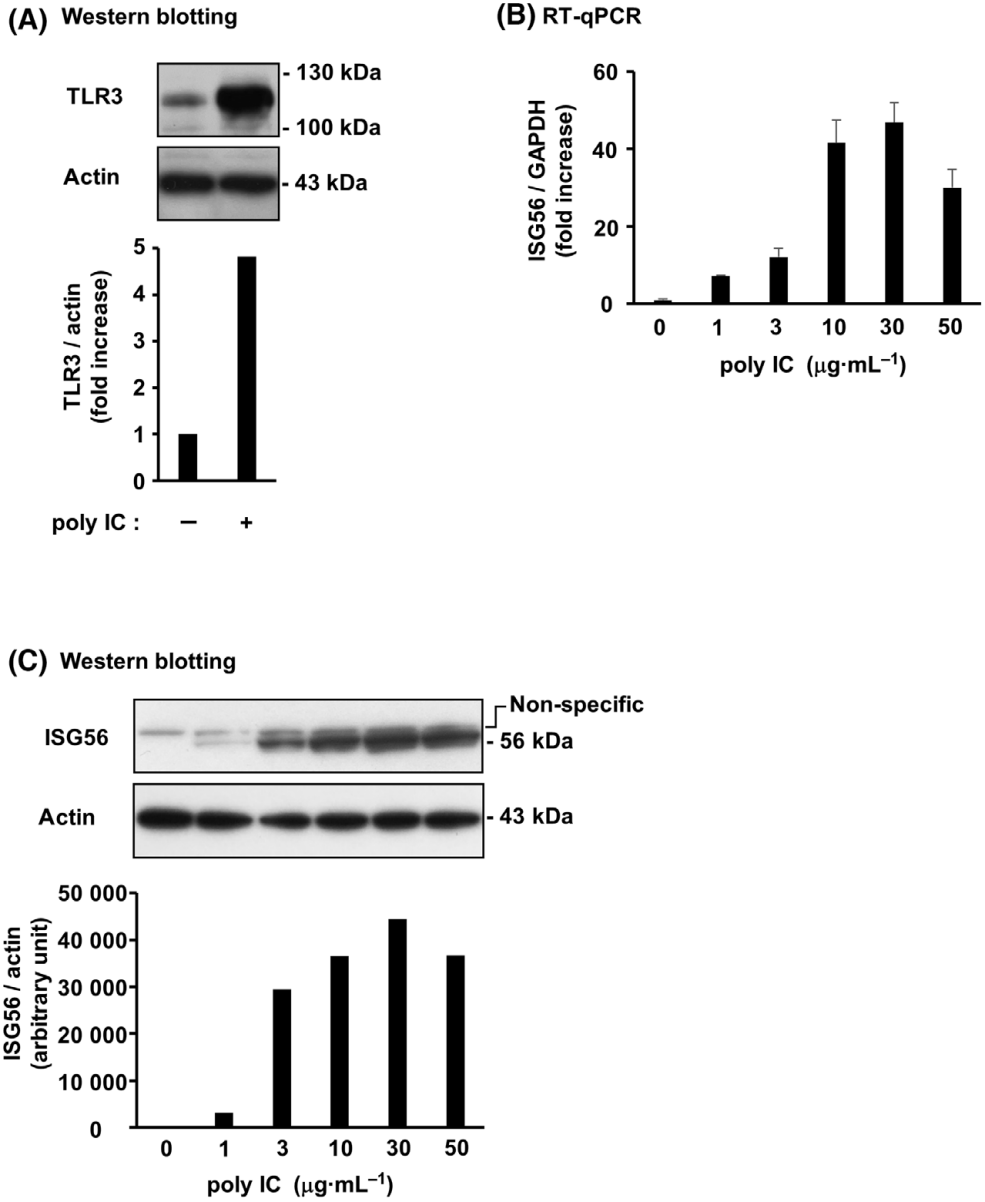
TLR3 was expressed in hRPTECs, and ISG56 was induced by poly IC, a TLR3 ligand, in a concentration‐dependent manner. After stimulation of cultured hRPTECs with poly IC (10 μg·mL^−1^) for 16 h, the cells were lysed, and the lysate was subjected to western blotting of TLR3 and actin (*n* = 1) (A). (B, C) Cultured cells were stimulated with various concentrations of poly IC. (B) After stimulating the cells with poly IC for 16 h, RNA extraction from the cells was performed to detect the expression of ISG56 and GAPDH mRNA by RT‐qPCR. The results are shown as the mean ± SD (*n* = 3). (C) After stimulating the cells with poly IC for 24 h, the cells were lysed and the lysate was used for western blotting of ISG56 and actin (*n* = 1).

**Fig. 3:**
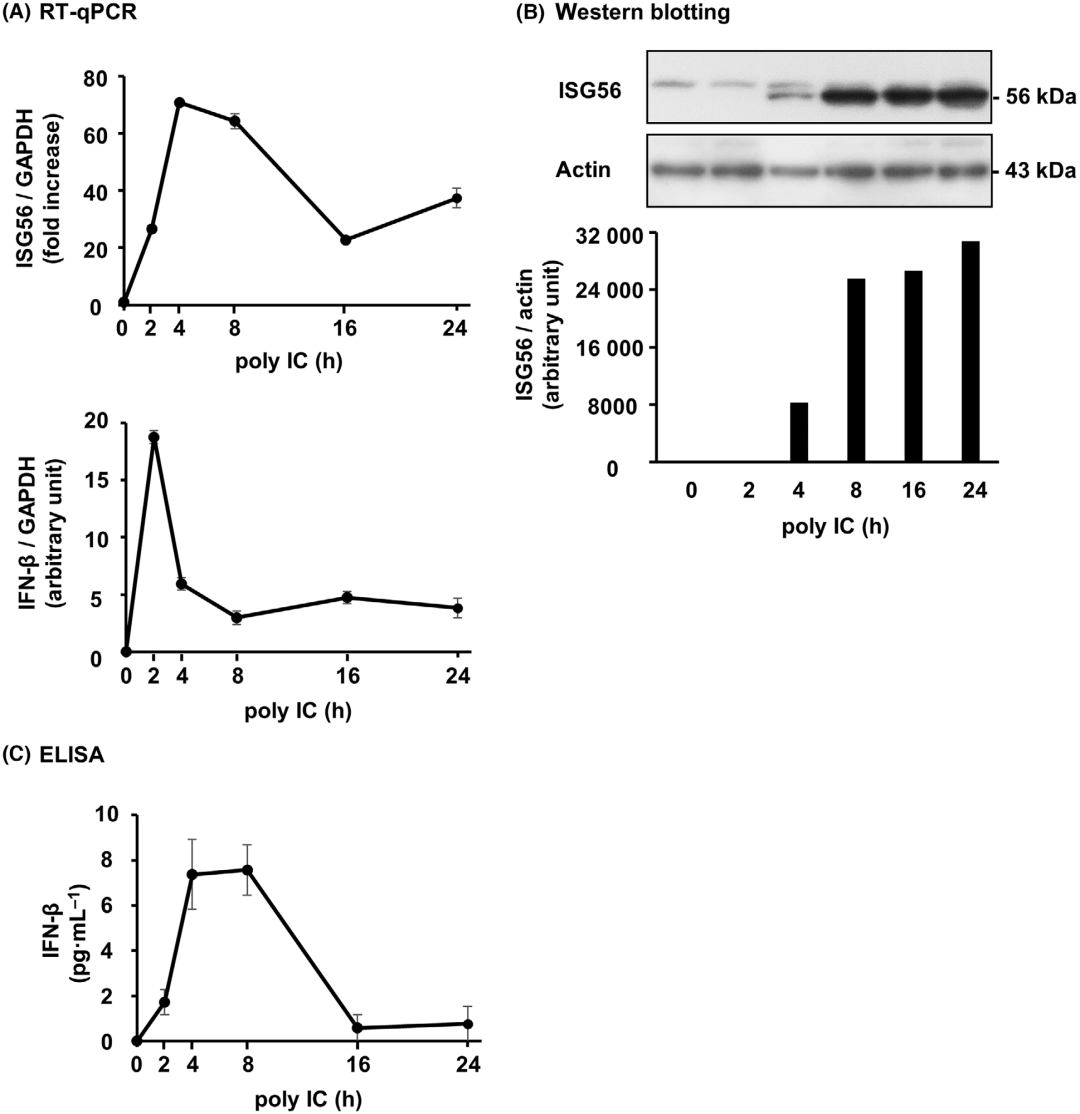
ISG56 and IFN‐β was expressed by poly IC depending on the time in hRPTECs. Poly IC (10 μg·mL^−1^) was used to stimulate cultured cells for up to 24 h. (A) RNA extraction from the cells was performed to detect of the expression of ISG56, IFN‐β, and GAPDH mRNA by RT‐qPCR. The results are shown as the mean ± SD (*n* = 3). (B) The cells were lysed and the lysate was used for western blotting of ISG56 and actin (*n* = 1). (C) An ELISA kit was used to measure the concentration of IFN‐β in the cell culture medium. The results are shown as the mean ± SD (*n* = 3).

TLR3, the receptor for poly IC, was constitutively expressed in unstimulated hRPTECs, and its expression was enhanced by poly IC (Fig. 2A). ISG56 expression was low in the unstimulated hRPTECs. ISG56 mRNA (Fig. 2B) and protein (Fig. 2C) expression was most elevated with stimulation of 10–30 and 10–50 μg·mL^−1^ poly IC, respectively. The cells were stimulated with 10 μg·mL^−1^ poly IC in the subsequent experiments. ISG56 mRNA expression peaked at 4 h, and then decreased (Fig. 3A). IFN‐β mRNA expression increased in advance of ISG56 mRNA and peaked at 2 h (Fig. 3A). The protein expression of ISG56 (Fig. 3B) and IFN‐β (Fig. 3C) peaked at 8–24 and 4–8 h, respectively.

### IFN‐β is involved in poly IC‐induced expression of ISG56 and CXCL10 in hRPTECs

**Fig. 4:**
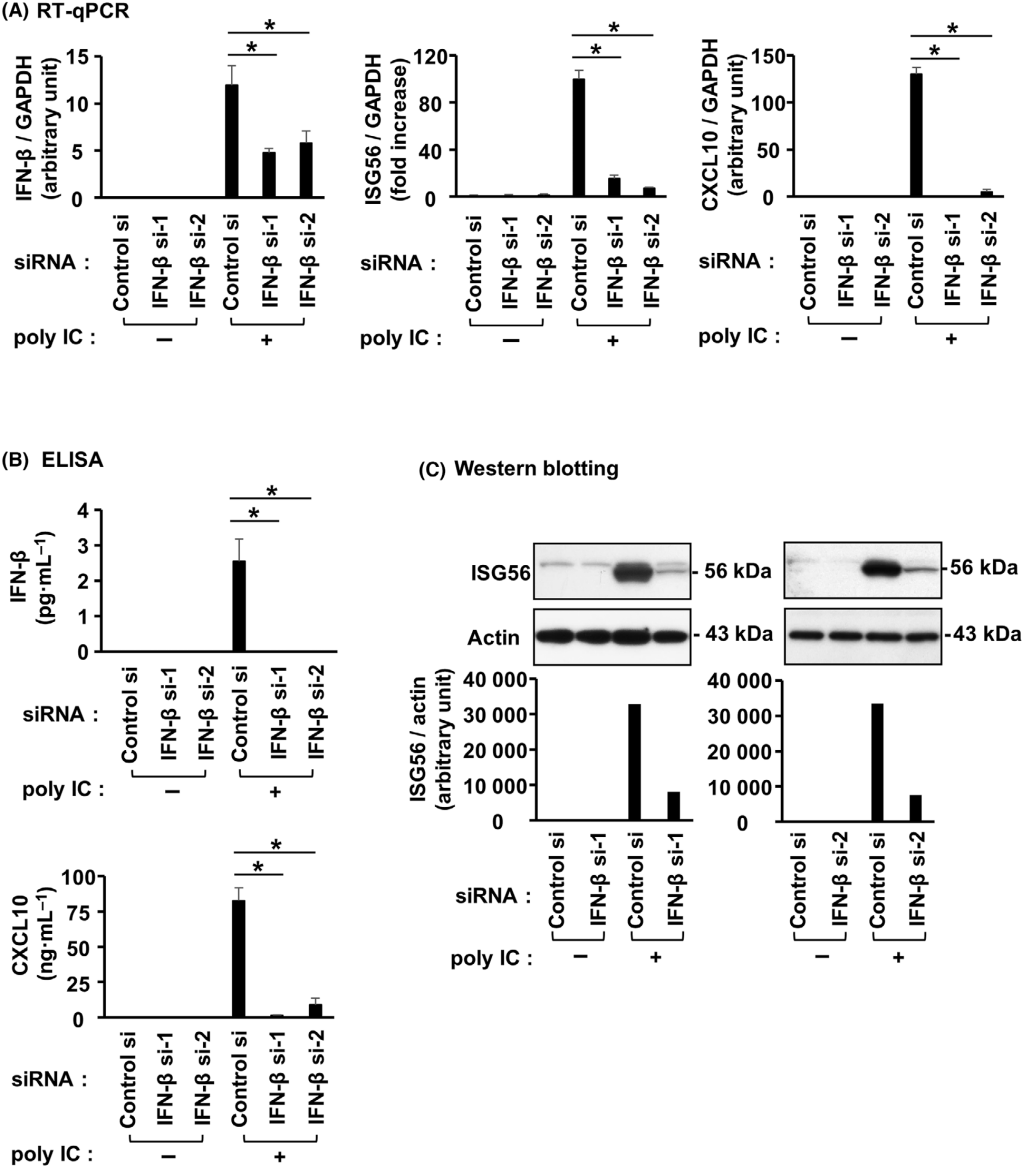
IFN‐β was engaged in ISG56 and CXCL10 induced by poly IC in hRPTECs. Control siRNA or two different siRNAs against IFN‐β were used to transfect cultured cells, and the cells were incubated for 24 h. Poly IC (10 μg·mL^−1^) was used to stimulate the cells. (A) After 2 h (for IFN‐β analysis) and 16 h (for other analysis) incubation, RNA extraction from the cells was performed, and IFN‐β, ISG56, CXCL10 and GAPDH expression was assayed by RT‐qPCR. The results are shown as the mean ± SD (*n* = 3). *P < 0.05 relative to cells transfected with control siRNA by a Mann–Whitney *U*‐test. (B) After 4 h (for IFN‐β analysis) and 24 h (for CXCL10 analysis) incubation, ELISA kits were used to measure the concentration of IFN‐β and CXCL10 in the cell culture medium. The results are shown as the mean ± SD (*n* = 3). *P < 0.05 relative to cells transfected with control siRNA by a Mann–Whitney *U*‐test. (C) After 24 h of incubation, the cells were lysed and the lysate was used for western blotting of ISG56 and actin (*n* = 1).

**Fig. 5:**
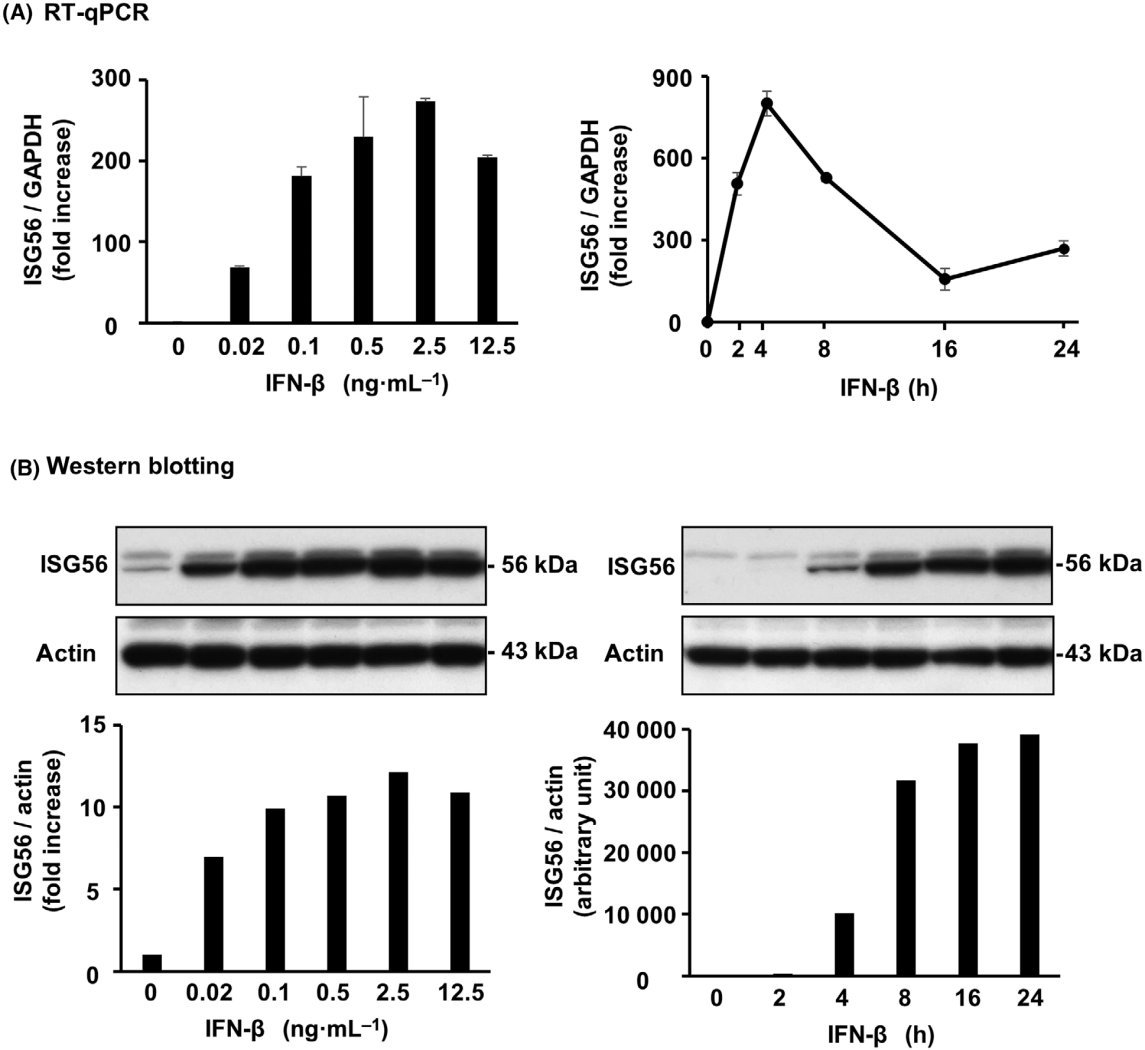
ISG56 was expressed by r(h)IFN‐β depending on the concentration and the time in hRPTECs. Cultured cells were treated with r(h)IFN‐β with various concentrations and for various hours. (A) RNA extraction from the cells was performed to detect of ISG56 and GAPDH mRNA expression by RT‐qPCR. The results are shown as the mean ± SD (*n* = 3). (B) The cells were lysed and the lysate was used for western blotting of ISG56 and actin (*n* = 1).

**Fig. 6:**
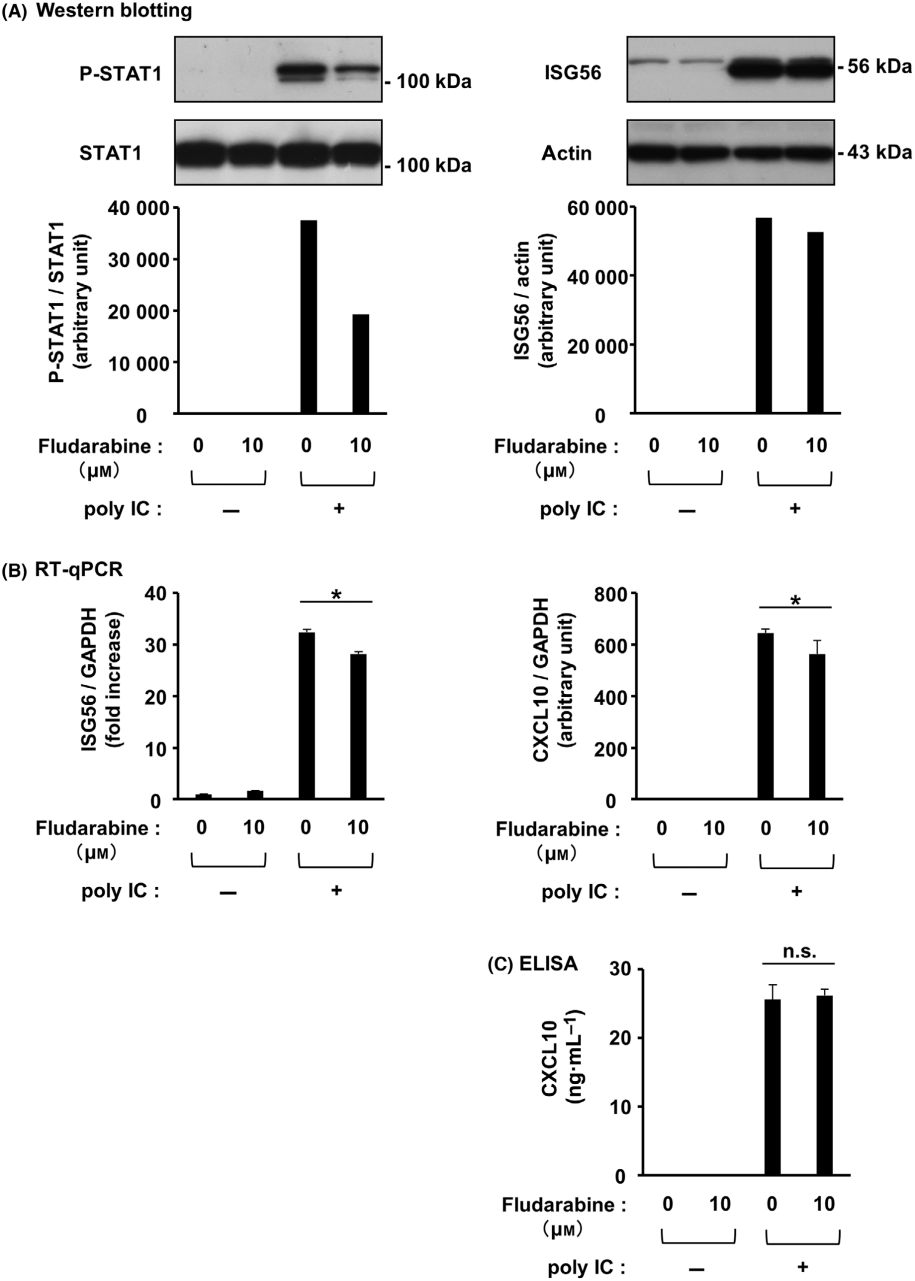
STAT1 was scarcely involved in ISG56 and CXCL10 induced by poly IC in hRPTECs. Fludarabine, an inhibitor of STAT1 activation, was added to cultured cells at a concentration of 10 μm, and the cells were incubated for 1 h. Poly IC (10 μg·mL^−1^) was used to stimulate the cells. (A) After 2 h (for phosphorylated STAT1 and STAT1 analysis) and 24 h (for ISG56 and actin analysis), the cells were lysed and the lysate was used for western blotting (*n* = 1). (B) After 16 h of incubation, RNA extraction from the cells was performed, and ISG56, CXCL10 and GAPDH mRNA expression was evaluated by RT‐qPCR. The results are shown as the mean ± SD (*n* = 3). *P < 0.05 relative to cells transfected with control siRNA by a Mann–Whitney *U*‐test. (C) After 24 h of incubation, the cell culture medium was collected, and an ELISA kit was used to measure the concentration of CXCL10. The results are shown as the mean ± SD (*n* = 3). *P < 0.05 relative to cells transfected with control siRNA by a Mann–Whitney *U*‐test. n.s., not significant.

Transfecting hRPTECs with two different IFN‐β siRNAs effectively suppressed the expression of IFN‐β mRNA (Fig. 4A) and protein (Fig. 4B). Upon IFN‐β knockdown, poly IC‐induced expression of ISG56 and CXCL10 mRNA (Fig. 4A) and protein (Fig. 4B,C) was significantly reduced. Next, we investigated the effect of r(h)IFN‐β‐stimulation on the expression of ISG56 in cultured cells. ISG56 mRNA (Fig. 5A) and protein (Fig. 5B) expression was most elevated with stimulation of 2.5 and 0.1–12.5 ng·mL^−1^ r(h)IFN‐β, respectively. The cells were stimulated with 0.1 ng·mL^−1^ r(h)IFN‐β in the subsequent experiments. ISG56 mRNA (Fig. 5A) and protein (Fig. 5B) expression peaked at 4 and 8–24 h, respectively. Pretreatment of cells with fludarabine, which effectively inhibited STAT1 activation (Fig. 6A), slightly reduced ISG56 and CXCL10 mRNA (Fig. 6B) but did not alter poly IC‐induced expression of ISG56 and CXCL10 proteins (Fig. 6A,C).

### ISG56 is involved in CXCL10 induction by poly IC or r(h)IFN‐β in hRPTECs

**Fig. 7:**
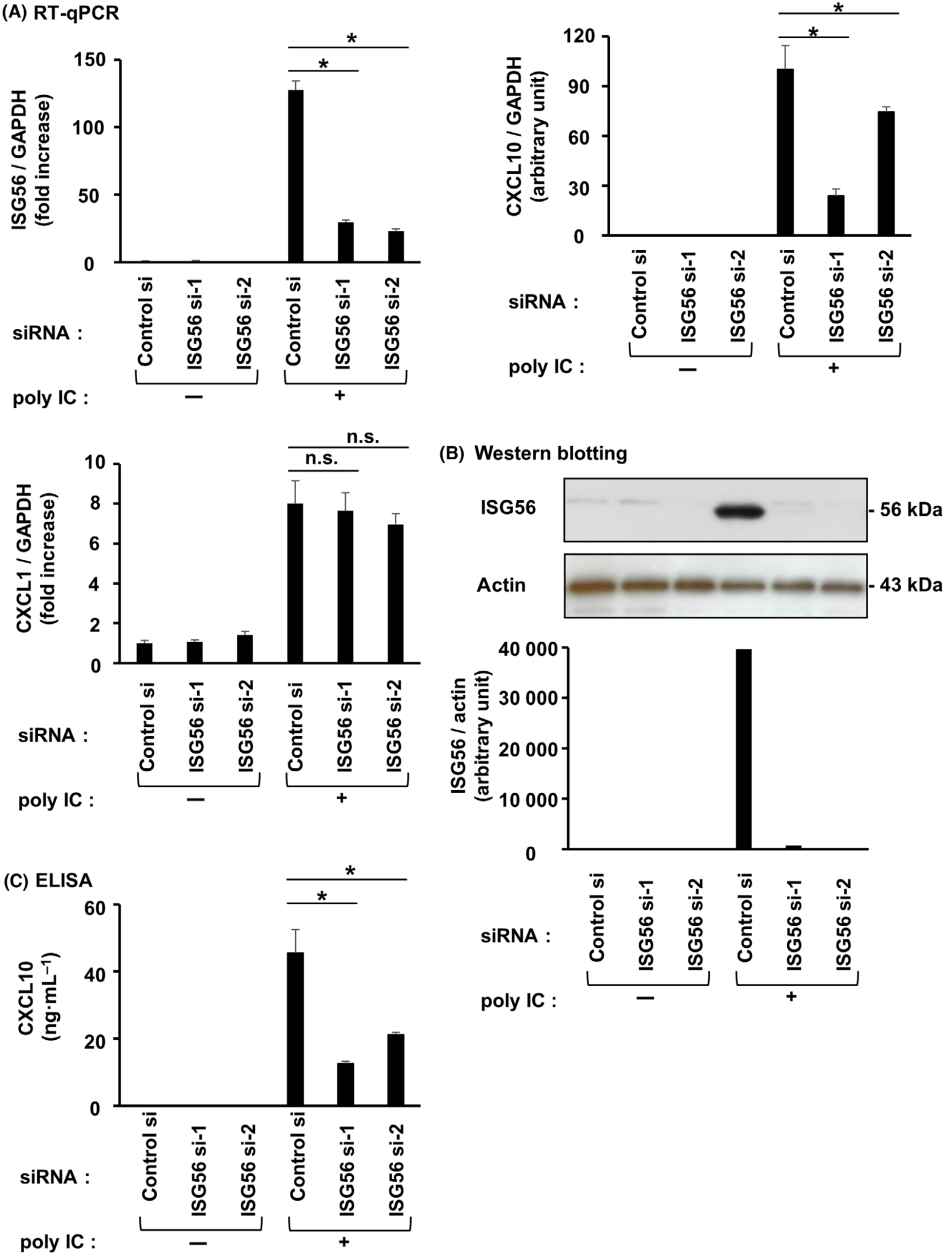
ISG56 was engaged in CXCL10 induced by poly IC in hRPTECs. Control siRNA or two different siRNAs against ISG56 were used to transfect cultured cells, and the cells were incubated for 24 h. Poly IC (10 μg·mL^−1^) was used to stimulate the cells. (A) After 16 h of incubation, RNA extraction from the cells was performed, and ISG56, CXCL10, CXCL1 and GAPDH expression was evaluated by RT‐qPCR. The results are shown as the mean ± SD (*n* = 3). *P < 0.05 relative to cells transfected with control siRNA by a Mann–Whitney *U*‐test. n.s., not significant. (B) After 24 h (for ISG56 analysis) of incubation, the cells were lysed and the lysate was used for western blotting of ISG56 and actin (*n* = 1). (C) After 24 h of incubation, an ELISA kit was used to measure the concentration of CXCL10 in the cell culture medium. The results are shown as the mean ± SD (*n* = 3). *P < 0.05 relative to cells transfected with control siRNA by a Mann–Whitney *U*‐test.

**Fig. 8:**
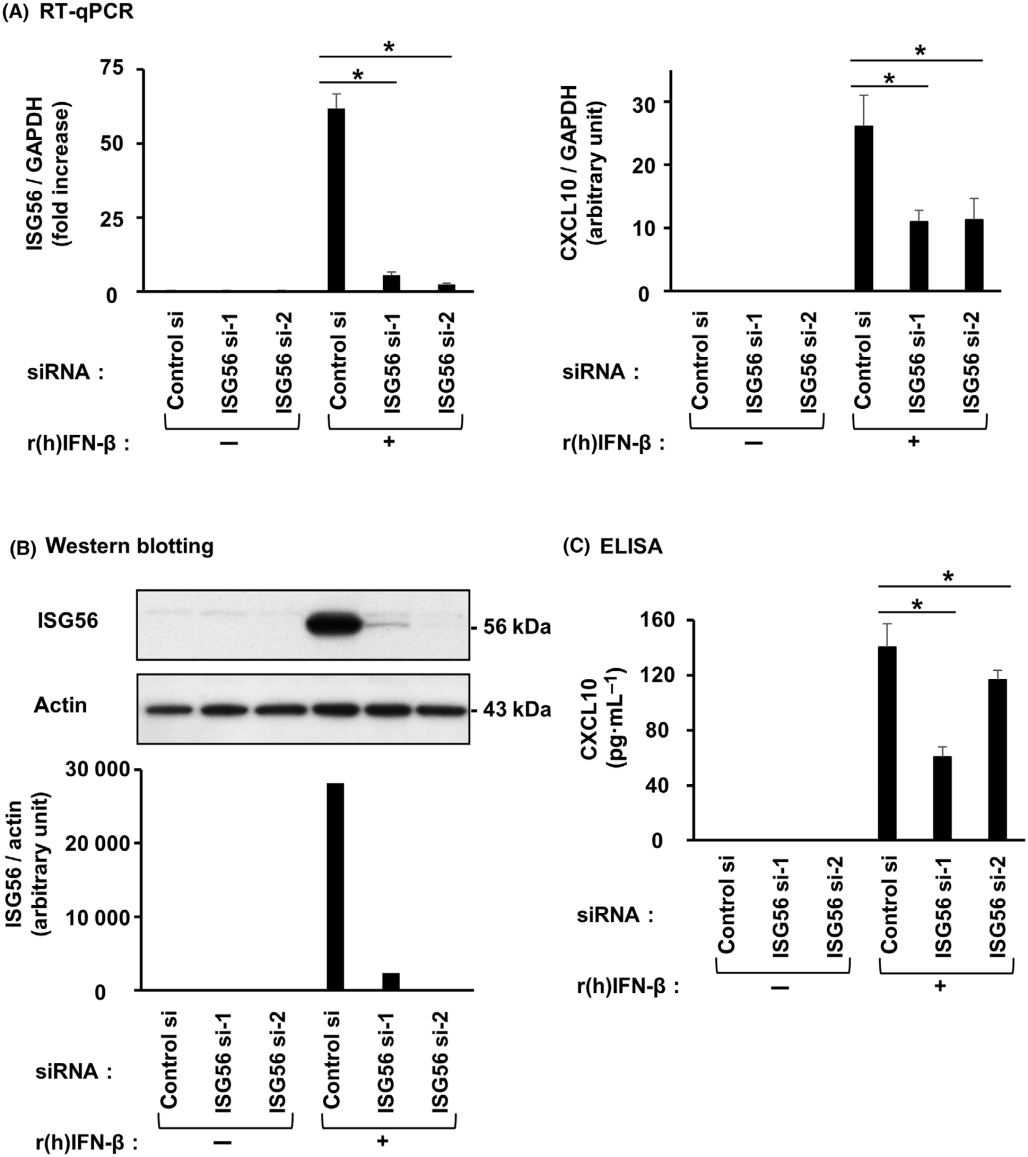
ISG56 was engaged in CXCL10 induced by IFN‐β in hRPTECs. Control siRNA or two different siRNAs against ISG56 were used to transfect cultured cells, and the cells were incubated for 24 h. r(h)IFN‐β (0.1 ng·mL^−1^) was used to stimulate the cells. (A) After 16 h of incubation, RNA extraction from the cells was performed, and ISG56, CXCL10 and GAPDH expression was evaluated by RT‐qPCR. The results are shown as the mean ± SD (*n* = 3). *P < 0.05 relative to cells transfected with control siRNA by a Mann–Whitney *U*‐test. (B) After 24 h (for ISG56 analysis) of incubation, the cells were lysed and the lysate was used for western blotting of ISG56 and actin (*n* = 1). (C) After 24 h of incubation, an ELISA kit was used to measure the concentration of CXCL10 in the cell culture medium. The results are shown as the mean ± SD (*n* = 3). *P < 0.05 relative to cells transfected with control siRNA by a Mann–Whitney *U*‐test.

Transfection of cells with two different ISG56 siRNAs effectively suppressed poly IC‐induced ISG56 mRNA (Fig. 7A) and protein (Fig. 7B) levels. Upon ISG56 knockdown, poly IC‐induced CXCL10 mRNA (Fig. 7A) and protein (Fig. 7C) levels were reduced. By contrast, knockdown of ISG56 did not alter poly IC‐induced mRNA expression of CXCL1 (Fig. 7A), a CXC motif chemokine that induces chemotaxis of neutrophils. Transfection of cells with ISG56 siRNAs also suppressed r(h)IFN‐β‐induced ISG56 mRNA (Fig. 8A) and protein (Fig. 8B). Upon knockdown of ISG56, r(h)IFN‐β‐mediated CXCL10 mRNA (Fig. 8A) and protein (Fig. 8C) were decreased.

### Immunofluorescence

**Fig. 9:**
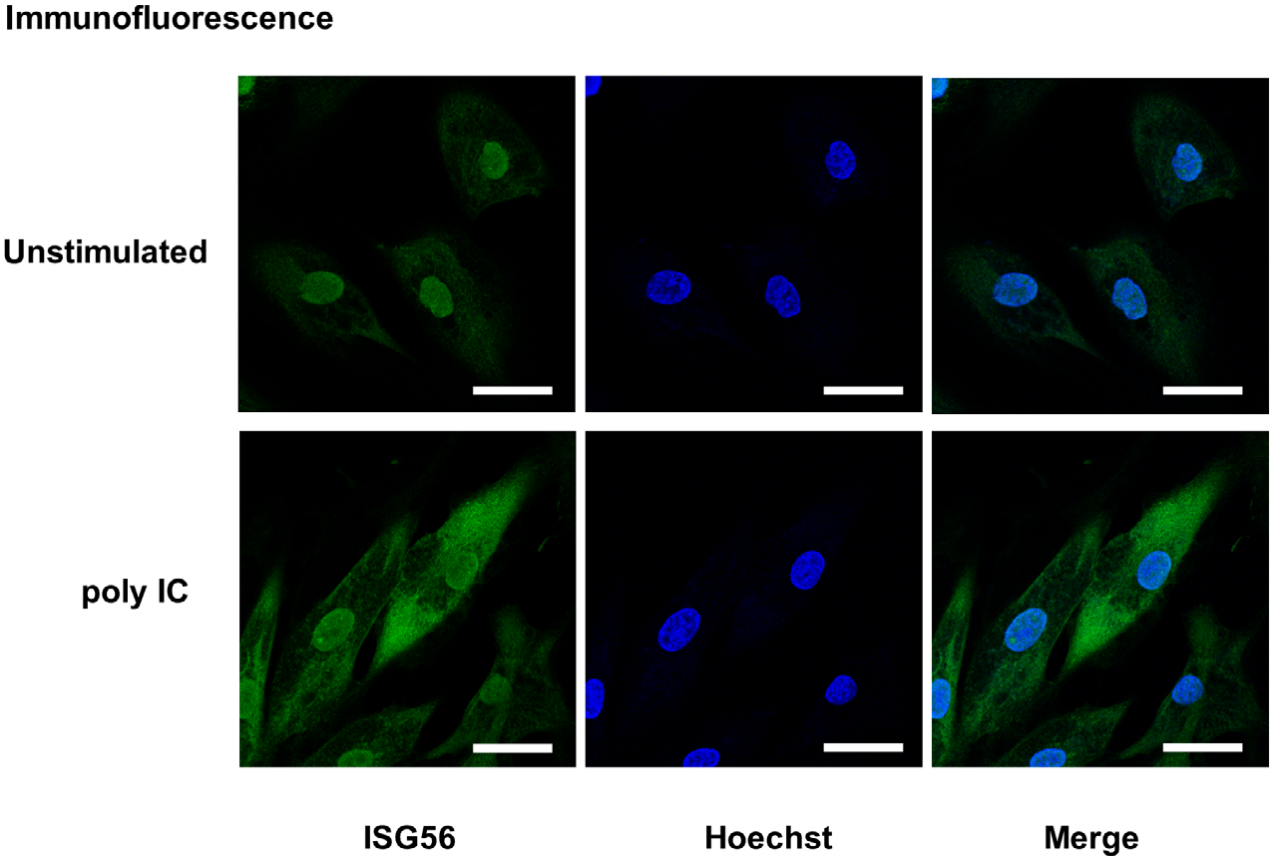
Intracellular localization of ISG56 protein in hRPTECs. The cells were stimulated with poly IC (10 μg·mL^−1^) for 24 h, fixed, permeabilized and incubated with an anti‐ISG56 antibody. Cell nuclei were stained with Hoechst 3334. Unstimulated cells and the cells after poly IC treatment were observed using laser scanning confocal microscopy. The white scale bar represents 50 μm (*n* = 1).

The intracellular localization of ISG56 protein in hRPTECs was analyzed by immunofluorescence. Faint immunoreactivity of ISG56 was found in nuclei of unstimulated cells. Upon poly IC stimulation, intense ISG56 immunoreactivity was observed in the cytoplasm (Fig. 9).

### ISG56 is involved in CXCL10 induction by JEV infection in hRPTECs

**Fig. 10:**
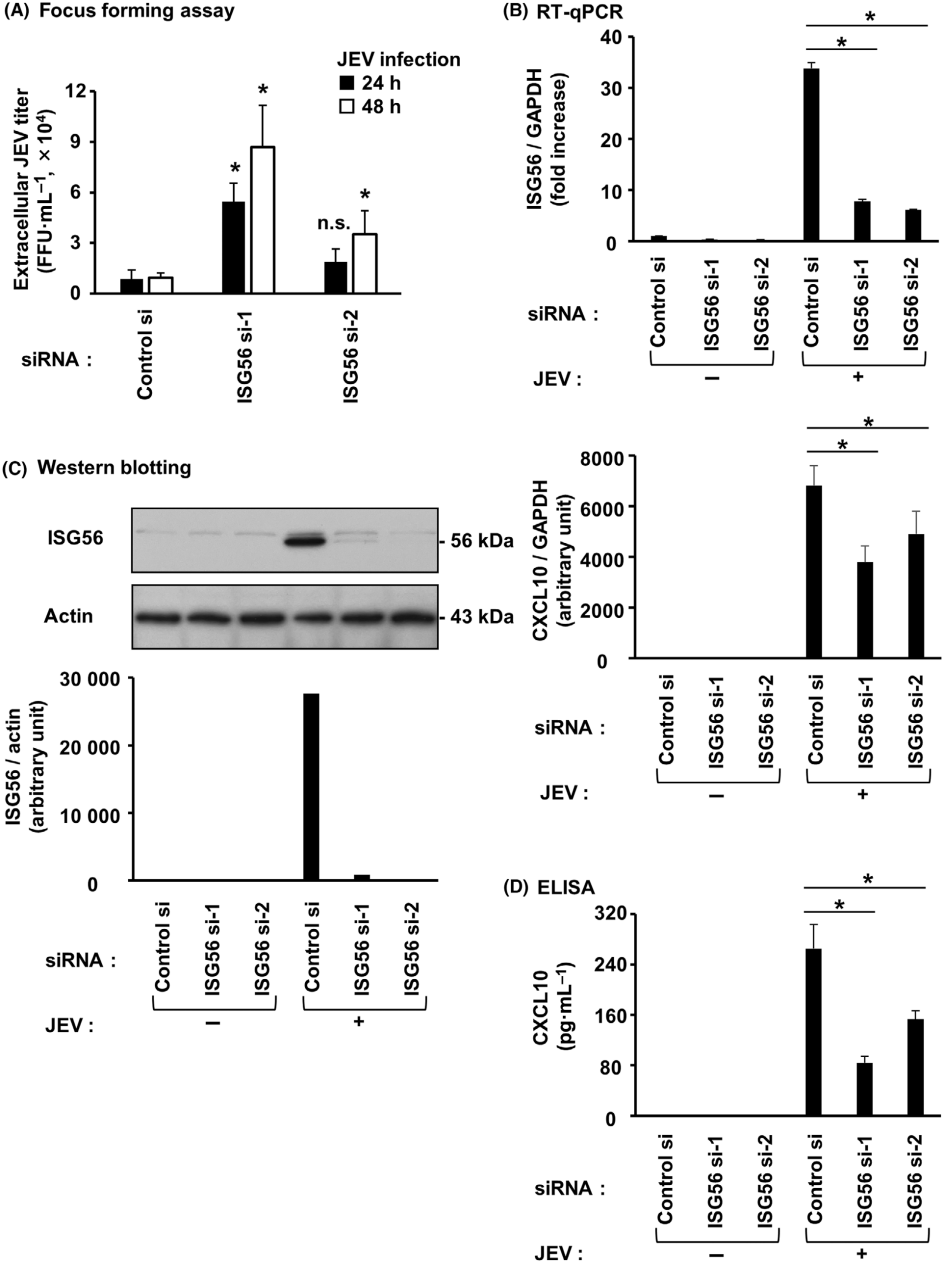
ISG56 was engaged in CXCL10 induced by JEV infection in hRPTECs. Cultured cells were transfected with control siRNA or two siRNAs against ISG56 and incubated for 24 h. JEV was added to each well at MOI = 1.0. (A) After 24 and 48 h of incubation, the medium was collected, and viral titers [focus forming unit (FFU)] were measured. The results are shown as the mean ± SD (*n* = 3). *P < 0.05 relative to cells transfected with control siRNA by a Mann–Whitney *U*‐test. n.s., not significant. (B) After 24 h of incubation, RNA extraction from the cells was performed, and ISG56, CXCL10 and GAPDH mRNA expression was evaluated by RT‐qPCR. The results are shown as the mean ± SD (*n* = 3). *P < 0.05 relative to cells transfected with control siRNA by a Mann–Whitney *U*‐test. (C) After 24 h of incubation, the cells were lysed and the lysate was used for western blotting of ISG56 and actin (*n* = 1). (D) After 24 h of incubation, the cell culture medium was harvested, and the concentration of CXCL10 was measured using an ELISA kit. The results are shown as the mean ± SD (*n* = 3). *P < 0.05 relative to cells transfected with control siRNA by a Mann–Whitney *U*‐test.

The cells were transfected with two different ISG56 siRNAs and then infected with JEV. Viral titers at 48 h after viral infection were significantly increased by knockdown of ISG56 (Fig. 10A). ISG56 mRNA (Fig. 10B) and protein (Fig. 10C) were induced by viral infection, and they were effectively suppressed by siRNAs. Knockdown of ISG56 reduced CXCL10 mRNA (Fig. 10B) and protein (Fig. 10D) levels upregulated by viral infection.

## Discussion

Morphological observation by phase contrast imaging revealed that hRPTECs formed monolayer and SEM analysis showed the characteristic morphology of renal tubular epithelial cells forming dense microvilli. The hRPTECs showed significant uptake of BSA‐Alexa488. In addition, hRPTECs expressed mRNA of cubilin and CD13, both of which are cellular markers of renal proximal tubular epithelial cells. These results suggest that the hRPTECs used in this study retained the characteristics of renal proximal tubular epithelial cells.

In this study, we found that poly IC treatment elevated ISG56 mRNA and protein expression in cultured hRPTECs. Next, because ISG56 is one of the ISGs, we investigated whether ISG56 expression is involved in IFN‐β. Observation of the time course showed that IFN‐β was quickly induced after poly IC treatment and was expressed before ISG56 expression. Knockdown of IFN‐β with siRNAs resulted in almost complete suppression of poly IC‐induced ISG56 and CXCL10 mRNA and protein. Furthermore, r(h)IFN‐β treatment also upregulated ISG56 mRNA and protein expression. In other words, the newly produced IFN‐β mediates the induction of ISG56 by autocrine or paracrine signaling. STAT1 plays a key role in the immune response against pathogens such as viruses and microbes by transmitting signals from IFNs [19]. In the present study, fludarabine, an inhibitor of STAT1 activation, scarcely affected ISG56 and CXCL10 expression induced by poly IC. This suggests that poly IC‐induced expression of ISG56 and CXCL10 is mostly independent of the STAT1 pathway in hRPTECs.

Next, we investigated the effects of ISG56 on poly IC‐induced CXCL10 expression. After the knockdown of ISG56 with siRNAs, CXCL10 mRNA and protein expression was partially attenuated. This suggests that ISG56 positively regulates TLR3‐mediated CXCL10 expression, in cooperation with other molecules. By contrast, for the same C‐X‐C motif chemokine, CXCL1, which mainly induces chemotaxis of neutrophils, knockdown of ISG56 did not affect poly IC‐induced CXCL1 mRNA expression. These findings indicate that the positive regulation of CXCL10 by ISG56 is specific. Furthermore, in viral infection experiments, knockdown of ISG56 resulted in increased viral titers, suggesting that ISG56 acts an antiviral molecule in hRPTECs. In addition, CXCL10 expression in cells infected with JEV was decreased by ISG56 knockdown, indicating that ISG56 induced not only by authentic dsRNA, but also by real virus is involved in CXCL10 expression.

CXCL10 is involved in the immune response to viral reactivation after renal transplantation and in renal rejection [20, 21]. Polyomavirus BK, which is estimated to infect more than 80% of the population [22], can be reactivated by immunosuppression after renal transplantation. In this case, CXCL10 acts antivirally by migrating lymphocytes and acting directly on the virus [14, 15]. However, upregulation of CXCL10 expression worsens prognosis of chronic graft nephropathy [21]. In a study on skin grafts, it was reported that neutralizing the chemokine CXCL9, which binds to CXCR3, as well as CXCL10, in the graft prolonged graft survival [23]. The main reason for the use of immunosuppressive drugs after renal transplantation is to prevent T cell‐mediated rejection. Therefore, expression of ISG56 may play a protective role against viral infection by increasing CXCL10 expression; however, the increase in CXCL10 that mediates the migration of lymphocytes may also induce rejection in renal transplantation. Another example is SARS‐CoV‐2 infection, which sometimes leads to acute kidney injury (AKI) and extensive renal tissue damage as a result of a cytokine storm and the direct action of the virus [24]. CXCL10 elevation correlates with the severity of SARS‐CoV‐2 infection and is a prognostic indicator [25]. In an *in vivo* study, deletion of microRNA‐146a, which regulates CXCL8 secretion in tubular cells, promoted the development of tubular lesions and renal fibrosis after AKI as a result of an uncontrolled cytokine storm [26]. Therefore, chemokines are double‐edged swords. Depending on the situation, low CXCL10 expression may not fulfill its antiviral function, while too much may cause tissue injury. Thus, CXCL10 expression may be regulated by multiple molecules.

**Fig. 11:**
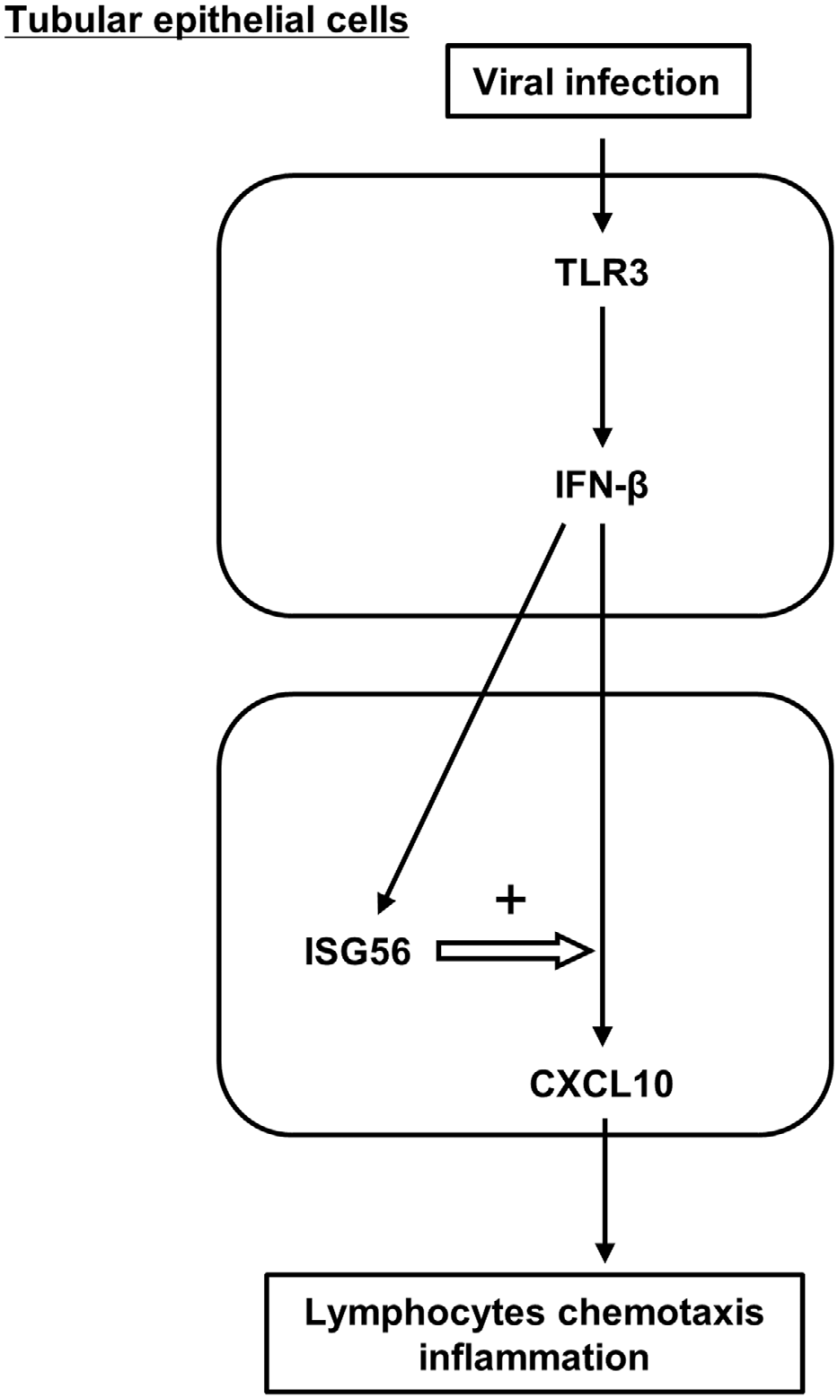
Schematic model of ISG56 function for TLR3/IFN‐β/CXCL10 in hRPTECs.

Taken together, we found that TLR3 signaling induces ISG56 expression via IFN‐β, and ISG56 positively regulates TLR3/IFN‐β‐mediated CXCL10 expression (Fig. 11). These results are consistent with previous observations in human mesangial [9] and glomerular endothelial cells [10]. ISG56, produced by various renal cells, may function together in physiological and pathological antiviral innate immunity. The expression and function of ISG56 on hRPTECs found in this study is novel and may provide a foundation for new treatment strategies to fight kidney diseases associated with viral infections.

However, this study has some limitations. The results were obtained from *in vitro* experiments. In complex *in vivo* mechanisms, not only immune responses mediated by TLR3, but also other signaling pathways may be activated, and there may be interactions between tubular epithelial cells and other cells. Finally, tetratricopeptide repeats function by binding to various proteins and RNAs [8]. However, this study did not elucidate the detailed molecular mechanisms by which ISG56 regulates CXCL10 expression. Further detailed studies should be conducted to determine the effects of ISG56 on CXCL10 expression following viral infections.

In summary, ISG56 was expressed by poly IC treatment, and IFN‐β‐mediated CXCL10 expression is positively regulated by ISG56 in hRPTECs. These findings suggest that ISG56 may play a critical role in the positive regulation of antiviral immune and inflammatory responses in hRPTECs.

## Conflicts of interest

The authors declare that they have no conflicts of interest.

### Peer review

The peer review history for this article is available at https://www.webofscience.com/api/gateway/wos/peer-review/10.1002/2211-5463.13851.

## Author contributions

MT and TI were responsible for all of the experiments and preparation of the manuscript. SS and EM contributed to the viral infection experiments. YK contributed to cell culture, RT‐qPCR and western blotting. YA contributed to SEM. SK and KS contributed to cell culture and the ELISA. MT, HT and TI contributed to the study design. All of the authors have read and approved the final version of the manuscript submitted for publication.

## Data Availability

The data generated in this study can be obtained from the corresponding author upon reasonable request.
